# Changing Educational Gradients in Life Expectancy With and Without Disease Among U.S. Older Adults From 2000 to 2010

**DOI:** 10.1093/geroni/igaa057.1630

**Published:** 2020-12-16

**Authors:** Matthew Farina, Phillip Cantu, Mark Hayward

**Affiliations:** 1 University of Texas at Austin, Austin, Texas, United States; 2 The University of Texas Medical Branch at Galveston, Galveston, Texas, United States

## Abstract

Recent research has documented increasing education inequality in life expectancy among U.S. adults; however, much is unknown about other health status changes. The objective of study is to assess how healthy and unhealthy life expectancies, as classified by common chronic diseases, has changed for older adults across education groups. Data come from the Health and Retirement Study and National Vital Statistics. We created prevalence-based life tables using the Sullivan method to assess sex-specific life expectancies for stroke, heart disease, cancer, and arthritis by education group. In general, unhealthy life expectancy increased with each condition across education groups. However, the increases in unhealthy life expectancy varied greatly. While stroke increased by half a year across education groups, life expectancy with diabetes increased by 3 to 4 years. In contrast, the evidence for healthy life expectancy provides mixed results. Across chronic diseases, healthy life expectancy decreased by 1 to 3 years for respondents without a 4-year degree. Conversely, healthy life expectancy increased for the college educated by .5 to 3 years. While previous research shows increases in life expectancy for the most educated, trends in life expectancy with chronic conditions is less positive: not all additional years are in lived in good health. In addition to documenting life expectancy changes across education groups, research assessing health of older adults should consider the changing inequality across a variety of health conditions, which will have broad implications for population aging and policy intervention.

